# Obstetric–Neonatal Care during Birth and Postpartum in Symptomatic and Asymptomatic Women Infected with SARS-CoV-2: A Retrospective Multicenter Study

**DOI:** 10.3390/ijerph19095482

**Published:** 2022-04-30

**Authors:** Rafael Vila-Candel, Víctor M. González-Chordá, Francisco Javier Soriano-Vidal, Enrique Castro-Sánchez, Noelia Rodríguez-Blanco, Ana Gómez-Seguí, Laura Andreu-Pejó, Cristina Martínez-Porcar, Carmen Rodríguez Gonzálvez, Patricia Torrent-Ramos, Nieves Asensio-Tomás, Yolanda Herraiz-Soler, Ramon Escuriet, Desirée Mena-Tudela

**Affiliations:** 1 Department of Nursing, Faculty of Nursing and Podiatry, Universitat de València, 46010 Valencia, Spain; rafael.vila@uv.es or; 2 Department of Obstetrics and Gynaecology, Hospital Universitario de la Ribera, 46600 Alcira, Spain; soriano_fravid@gva.es; 3 Foundation for the Promotion of Health and Biomedical Research in the Valencian Region (FISABIO), 46020 Valencia, Spain; 4 GIENF-281 Nursing Research Group, Nursing Department, Univesitat Jaume I, 12006 Castelló de la Plana, Spain; pejo@uji.es (L.A.-P.); dmena@uji.es (D.M.-T.); 5 Department of Obstetrics and Gynaecology, Hospital Lluis Alcanyís, 46800 Xàtiva, Spain; 6 College of Nursing, Midwifery and Healthcare, University of West London, London TW8 9GB, UK; enrique.castrosanchez@uwl.ac.uk; 7 Health Protection Research Unit, Healthcare-Associated Infections and Antimicrobial Resistance, Imperial College London, London SW7 2BX, UK; 8 Department of Nursing, Universidad CEU Cardenal Herrera, Plaza Reyes Católicos, 19, 03204 Elche, Spain; noelia.2ww@gmail.com; 9 Department of Obstetrics and Gynaecology, Hospital Marina Baixa, 03570 Villajoyosa, Spain; 10 Department of Obstetrics and Gynaecology, Hospital Universitario y Politécnico La Fe, 46026 Valencia, Spain; anagomezsegui@gmail.com (A.G.-S.); niasto@hotmail.com (N.A.-T.); 11 Department of Paediatrics, Hospital Universitario de la Ribera, 46600 Alzira, Spain; cmartinezporcar@gmail.com; 12 Department of Obstetrics and Gynaecology, Hospital Universitario de Vinalopó, 03293 Elche, Spain; matrona.haptonomia@gmail.com; 13 Preventive Medicine Service, Hospital General de Castellón, 12071 Castelló de la Plana, Spain; ptorrent@uji.es; 14 Nursing Department, Univesitat Jaume I, 12006 Castelló de la Plana, Spain; 15 Department of Obstetrics and Gynaecology, Consorcio Hospital General Universitario Valencia, 46014 Valencia, Spain; yolandaherraiz@hotmail.com; 16 Facultat d’Infermeria i Podologia, Universitat de València, 46100 Valencia, Spain; 17 Ghenders Research Group, School of Health Sciences Blanquerna, Universitat Ramon Lull, Carrer Padilla 326, 08025 Barcelona, Spain; rescuriet@gencat.cat; 18 Catalan Health Service, Government of Barcelona, Travessera de les Corts 131, 08028 Barcelona, Spain

**Keywords:** COVID-19, SARS-CoV-2, obstetric nursing, neonatal nursing, labour, obstetric

## Abstract

This study analyses the obstetric–neonatal outcomes of women in labour with symptomatic and asymptomatic COVID-19. A retrospective, multicenter, observational study was carried out between 1 March 2020 and 28 February 2021 in eight public hospitals in the Valencian community (Spain). The chi-squared test compared the obstetric–neonatal outcomes and general care for symptomatic and asymptomatic women. In total, 11,883 births were assisted in participating centers, with 10.9 per 1000 maternities (*n* = 130) infected with SARS-CoV-2. The 20.8% were symptomatic and had more complications both upon admission (*p* = 0.042) and during puerperium (*p* = 0.042), as well as transfer to the intensive care unit (ICU). The percentage of admission to the Neonatal Intensive Care Unit (NICU) was greater among offspring of symptomatic women compared to infants born of asymptomatic women (*p* < 0.001). Compared with asymptomatic women, those with symptoms underwent less labour companionship (*p* = 0.028), less early skin-to-skin contact (*p* = 0.029) and greater mother–infant separation (*p* = 0.005). The overall maternal mortality rate was 0.8%. No vertical transmission was recorded. In conclusion, symptomatic infected women are at increased risk of lack of labour companionship, mother–infant separation, and admission to the ICU, as well as to have preterm births and for NICU admissions.

## 1. Introduction

Disease due to severe acute respiratory syndrome coronavirus-2 (SARS-CoV-2) infection had caused over 4.7 million deaths worldwide by 2021 [1]. Although most countries adopted measures to contain the pandemic, including lockdowns and preventive hygiene protocols [2] as well as vaccination programs wherever vaccines where available [3], SARS-CoV-2 infection remains a significant global health threat.

With regard to coronavirus disease-2019 (COVID-19), pregnant women do not seem to be more susceptible to infection than the general population [4]. However, in the event of disease symptoms that appear to be more severe, particularly during the third trimester of pregnancy, there are more frequent admissions to the Intensive Care Unit (ICU) and a greater risk of poorer maternal and neonatal health outcomes (such as preterm birth, caesarean sections, and low birth weight) [5,6,7,8].

Routine evidence-based clinical practices of benefit for maternal and childbirth care (labour companionship, early skin-to-skin contact, breastfeeding, and rooming-in in maternity) have been modified or interrupted during the pandemic, which could reduce the quality of birth care models [9,10]. Furthermore, vertical transmission seems possible [11], although the mechanisms of such transmission remain unclear [12,13]. Whilst severe events for newborn infants seem rare [13], perhaps thanks to the passive transmission of anti-SARS-CoV-2 antibodies via the transplacental route and in breast milk [14], there may still be concern about these potential effects [15,16].

The primary aim of this study was to analyse the obstetric–neonatal outcomes of women in labour with symptomatic and asymptomatic SARS-CoV-2 infection. The study also aimed to describe and contrast the routine care received by these groups of women and their newborns.

## 2. Materials and Methods

### 2.1. Study Population and Sampling Criteria

A retrospective, multicenter, observational study was carried out based on the review of the clinical records of pregnant women assisted during labour and birth, with a positive real-time polymerase chain reaction (RT-PCR) test result for SARS-CoV-2 in nasopharyngeal exudate at the time of admission. The study was carried out in eight state-funded hospitals of the Valencian community in Spain. These hospitals (four in Valencia, one in Castellón, three in Alicante) were all reference centers for their province, and cared for at least 1000 births per year, or were located in rural areas with large catchment populations. Overall, the participating hospitals served one million people and attended ~12,000 births in the previous year. The information about the study was disseminated via the regional research network and suitable health care organizations approached by the researchers. The study period was from 1 March 2020 to 28 February 2021.

The study population comprised women giving birth in any of the participating hospitals. The inclusion criteria were: (a) women with a positive RT-PCR test for SARS-CoV-2 RNA in nasopharyngeal exudate performed on hospital admission for labour and birth; and (b) infants born of infected mothers, with RT-PCR testing for SARS-CoV-2 in nasopharyngeal exudate during hospital admission (<48 h, not extracted from the placenta or amniotic fluid).

Pregnant women infected with SARS-CoV-2 and admitted to the hospital for medical reasons other than birth were excluded from the study.

### 2.2. Measurements

The research staff at each participating center reviewed the obstetric history, neonatal and postpartum outcomes, and general labour care records of all the positive patients assisted during the study period. The variables related to birth and postpartum were collected from the Orion Logis^®^ electronic health records, while the data for mothers and newborns related to follow-up during the first six weeks after birth were obtained from the Abucasis II^®^ health database. Any readmission via the emergency room of mothers or newborns registered at the same hospitals were identified during the 6 weeks from the Abucasis II^®^ database. Both electronic medical records are routinely used by all the health facilities in the Valencian Community, including all the centers participating in the study. The following variables were collected:

- Demographic variables: maternal age, country of origin, and the hospital where the birth occurred.
- Obstetric–neonatal variables: Gestational age at the time of birth, parity (primiparous/multiparous), previous maternal history of health problems (diabetes mellitus/hypertension/cardiac diseases/neurological diseases/thrombotic diseases/thyroid diseases/drugs misuse/COVID-not related infection diseases), gestational disease (preeclampsia/eclampsia/gestational hypertension/gestational diabetes/hypothyroidism/hyperthyroidism/COVID-not related infectious diseases), fetal alterations identified by health care providers (preterm birth, small for gestational age/large for gestational age/fetal growth restriction/congenital abnormality), start of labour (spontaneous/induced/elective caesarean section), type of birth (eutocic, instrumental birth/caesarean section [CS]), cause of CS, maternal complications prior labour and during puerperium (respiratory, cardiac, neurologic, thrombotic manifestations, and COVID-related coagulopathy), maternal admission to the intensive care unit (ICU) prior to birth and/or puerperium, cause of maternal admission to the ICU, and Apgar score at one and five minutes. Mother to child transmission can occur in different stages, including in utero, intrapartum, or postnatal. Our study defined vertical transmission in the early postnatal period (<48 h) as a positive test for SARS-CoV-2 maternal infection at admission, coupled with a confirmed positive test for the newborn [17], infant admission to the neonatal ICU (NICU), and cause of admission to the NICU.
- Symptomatic with SARS-Cov-2: Participants with SARS-CoV-2 infection that present symptoms such as fever, cough, shortness of breath, fatigue, body aches, headache, anosmia, ageusia, nausea or vomiting, and diarrhea.
- Asymptomatic with SARS-Cov-2: Participants with SARS-Cov-2 infection but who did not develop compatible clinical manifestations.
- Clinical variables: Time of result of the RT-PCR SARS-CoV-2 test for the mother (ante-, intra-, or postpartum), the result of the RT-PCR SARS-CoV-2 test for the newborn infant within 48 h (positive or negative), and follow-up of complications and readmissions (mother/newborn infant, with reasons) during the first six weeks after birth. Maternal or newborn death (up to 28 days) if COVID-related.
- Obstetric and neonatal general labour care: Late clamping (clamping and cutting the cord at least one minute from birth, or when the umbilical cord stopped pulsating [18]), early skin-to-skin contact [SSC] (defined as prone placing of the naked infant on the mother’s bare chest at birth, in the first minute after birth, or very soon afterwards [19]), labour companionship, mother–infant separation during hospital admission, and the reason for separation.
- Breastfeeding-related variables: Type of feeding at discharge and 6 weeks postpartum (exclusive breastfeeding, formula feeding, mixed feeding). Exclusive breastfeeding (EBF) was defined as offering only breast milk and excluding all other food or fluids, including water. This case definition did, however, allow the infant to receive oral rehydration salts, drops, and syrups (vitamins, minerals, and medicines) [20,21]. Mixed feeding was defined as the combination of breastfeeding and formula feed.

Obstetric and neonatal outcomes were compared between symptomatic and asymptomatic SARS-CoV-2 infected women.

- The obstetric outcomes were: Preterm birth < 37 weeks; foetal growth restriction (birth weight percentile < 5); induced hypertension in pregnancy; gestational diabetes; CS; operative birth; ICU admission; maternal complications prior labour/during puerperium COVID-related: cardiac, neurologic, thrombotic, and respiratory manifestations; and maternal death.

The neonatal outcomes were: small for gestational age (birth weight below the 10th percentile for babies of the same gestational age); large for gestational age (birth weight beyond 90th percentile for babies of the same gestational age); Apgar < 7 at 5 min; and NICU admission.

The obstetric and neonatal general care were compared between symptomatic and asymptomatic SARS-CoV-2-infected women were: a labour companion; late umbilical cord clamping; early SSC; mother–infant separation; and EBF (at discharge/6 weeks).

A common database was created through REDCap^®^, where all the researchers responsible for each hospital were entering the data for later analysis.

### 2.3. Statistical Analysis

Basic descriptive statistics were expressed as the mean ± standard deviation (SD) in the case of continuous variables, and as frequencies and percentages in the case of categorical variables. Normal data distribution was assessed using the Kolmogorov–Smirnov test.

The incidence rate was determined using the total number of new positive SARS-CoV-2 cases per 1000 maternities and divided by the number of births during the study period. The chi-squared test was used to compare the obstetric–neonatal general care rates according to the different qualitative variables, corrected by the Fisher exact test. Likewise, the chi-squared test and crude odds ratio (OR) with 95% confidence intervals (CIs) were used to compare the different study variables between asymptomatic SARS-CoV-2 positive women and women who developed COVID-19 (symptomatic cases). Statistical significance was considered for *p* < 0.05. Data analysis was performed using the R statistical package (version 4.0.5, R Foundation for Statistical Computing, Vienna, Austria).

### 2.4. Ethical Considerations

Patients were not involved in the development of the research questions, the study’s design, or the recruitment of participants. Due to the retrospective study design, patient informed consent was not required. All patient data were handled anonymously. The local ethics committees approved the study protocols in the eight centers. The ethical principles of medical research contemplated by current Spanish legislation have been considered, and the study was conducted following the Declaration of Helsinki.

## 3. Results

In total, 11,883 births were assisted during the study period in the participating hospitals. We included all women with SARS-CoV-2 infection admitted to the participating hospitals during the study period (130; 10.9 per 1000 maternities). There was no loss of any participant. The case distribution according to hospitals is shown in Table 1. Hospital H4 had an incidence of 17.8 per 1000 maternities, and hospital H8 assisted the largest number of positive cases. The majority of women were born in Spain (53.8%), with a mean age of 32 ± 5.1 years. There were statistically significant differences between symptomatic and asymptomatic women for any maternal age (Table 2).

Regarding the women’s characteristics, 50% were primiparous, with a mean gestational age of 39.2 ± 1.6 weeks, and most had no previous maternal, gestational, or foetal disease conditions, without significant differences between symptomatic and asymptomatic women (*p* < 0.05). Positivity for SARS-CoV-2 was confirmed before or during labour in 91.5% of cases, and 8.5% after birth; 56.2% had laboured spontaneously, with an overall induction rate of 39.2%. Induction of labour was higher, but without significant differences, among symptomatic women (44.4% vs. 37.9%; *p* = 0.09). The 4.6% of women had an elective CS, without significant differences between symptomatic and asymptomatic women (*p* = 0.09). There were no statistically significant differences between symptomatic and asymptomatic women in terms of maternal (*p* = 0.084), gestational (*p* = 0.089), or fetal disease conditions (*p* = 0.719).

In terms of the clinical parameters, 20.8% of women had symptoms upon admission, fever and cough (22.2% each) and headache (18.5%) being the most frequent manifestations. Other symptoms were 11.1% anosmia, 11.1% dyspnoea, 3.7% vomiting, 3.7% ageusia, and 7.4% body aches. Prior to birth, 7.4% of symptomatic women were admitted to the ICU with COVID-related respiratory distress versus none of the asymptomatic women (*p* = 0.042); of these women, 3.7% required mechanical ventilation due to dyspnoea and COVID-related thrombotic stroke, while 7.4% of symptomatic women were admitted to the ICU during the postpartum period compared with 1.9% asymptomatic women (*p* = 0.042). Mechanical ventilation was not required for any asymptomatic women admitted to the ICU. The admissions were not COVID-related (one preeclampsia, and one postpartum haemorrhage). However, both symptomatic women required mechanical ventilation. The mortality rate was 0.8%; among all participants, death was recorded in a woman with symptoms (specific mortality rate = 3.7%) (Table 3).

None of the newborn infants tested positive for SARS-CoV-2 infection during hospital admission. Organizational issues caused the reasons for admission of newborns to the NICU before discharge (19.2%), and none of them concerned a disease associated with maternal COVID-19. The reasons were separation following protocol active on the date (26.9%), prematurity (23.1%), stabilization to maladaptation after birth (15.4%), hyperbilirubinemia (11.5%), social services/adoption (7.6%), 3.8% sepsis (3.8%), observation following cerebral-vascular event (3.8%), and maternal drug misuse (3.8%).

The obstetric and neonatal general care is presented in Table 4. Early SSC occurred in 75.4% of births, and late clamping was performed in 55.6%. Mother–infant separation occurred in 19.2% of the cases, with no clinical indication in 5.4% and with a maternal accompaniment rate of 53.8%. On the other hand, asymptomatic women had a greater percentage of early SSC (79.6% vs. 59.3%; *p* = 0.029), a greater labour companion rate (64.1% vs. 40.7%; *p* = 0.028), and less mother–infant separation (13.6% vs. 40.7%; *p* < 0.001). There were no statistically significant differences in late umbilical cord clamping (asymptomatic = 57.3%; symptomatic = 40.7%; *p* = 0.125).

Concerning EBF, the percentage at the time of hospital discharge was 70.8%. The EBF rate at six weeks after birth was 53.1%. We recorded no statistically significant differences in the type of feeding at hospital discharge or the type of feeding at six weeks after birth, between women with and without COVID-19 symptoms.

Table 5 presents the obstetric–neonatal results and care received during labour, birth, and puerperium of women infected with SARS-CoV-2. There were differences between symptomatic and asymptomatic participants. The symptomatic women were nine times more likely to have a preterm birth (95% CI: 2.2–41.2) and eight times more likely to be admitted to the ICU (95% CI: 1.5–50.9); their infants were five times more likely to be admitted to the NICU (95% CI: 1.9–13.8).

Regarding general care for symptomatic women, they were more likely to be unaccompanied during labour (95% CI: 1.1–4.2), also markedly likely to experience mother–child separation (95% CI: 1.57–10.36) and twice as likely to experience non-performance of early SSC (95% CI: 1.1–4.1) compared to asymptomatic women.

CS was significantly associated to preterm birth (OR = 12.2; 95% CI: 2.8–53.6), admission to the ICU (OR = 11.1; 95% CI: 1.9–64.7), and admission to the NICU (OR = 5.6; 95% CI: 2.0–15.2). Additionally, a mother with preterm birth was associated with ICU admission (OR = 19.7; 95% CI: 3.3–118.8); data are not shown.

## 4. Discussion

This study identified an increased risk of adverse obstetric–neonatal outcomes (preterm birth and ICU/NICU admission) and poorer obstetric–neonatal general care (mother–infant separation, not early SSC, lack of a labour companion) among symptomatic SARS-CoV-2 infected pregnant women versus those without symptoms of the infection.

In our study, the incidence of infection among women in labour was low (10.9 per 1000 maternities). A possible cause could be due to the fact that the fear, anxiety, depression, or other psychopathological disorders that pregnant women may possess, for those who have been able to live during the pandemic, they have developed a self-confinement mechanism to avoid infection [22,23], although these hypotheses should be confirmed in future studies. In addition, in our study, only 20.8% of women in the study developed symptoms consistent with COVID-19. Other reports have varied with between 21% to 26% [21,24]. The most common symptoms reported were fever, headache, and cough, again in common with other multinational studies [8,21,25,26,27].

### 4.1. Obstetrical–Neonatal Outcomes

In our series, the induction rate (37.9%) was significantly higher among symptomatic women and higher than other series (22.3%) [8]. This was possibly related to patient care adjustments made by the clinical teams at the labour wards of the different hospitals based on available human and material resources [10].

It is reported in a living systematic review that SARS-CoV-2 infection was associated with increased risk of preterm birth [24,28], in line with our results that shown a nine-fold increased risk in symptomatic women.

With an observed increase in the probability of adverse outcomes in symptomatic versus asymptomatic women, the recorded incidence of CS was lower than reported elsewhere, which has varied between 45% to 60% [24,29,30,31,32]. This low incidence of CS in our series, compared to other studies [2,8,32], may be explained by other factors. Healthcare professionals may have adopted a more expectant attitude in childbirth, driven by a lack of knowledge of this new disease, and the risk and concern about infection among professionals [33,34,35]. Nevertheless, we found an association between CS and admission to the ICU and NICU [5,36]. This may reflect an association towards the indirect impact of SARS-CoV-2 on maternity care and perinatal morbidity [9,24,29].

Even though the percentage of complications was low, as per other studies (5.5–8.4%) [8,24], the risk of an adverse outcome in the prepartum and/or postpartum period increased in symptomatic patients, especially those with severe disease. In addition, as other studies have shown [21,28,34,37], the maternal mortality rate attributed to COVID-19 was higher than our study (~1.6% vs. 0.8%). Our study identified no association between maternal background disease and the manifestation of COVID-19 symptoms. However, even with a lower reported ICU admission rate (3.7%, 4/130) than other studies (8.4–9.6%) [8,11,28], maternal complications associated with the infection and required admission to the ICU were only observed in symptomatic women, with pneumonia and thrombotic stroke being the most frequent conditions, in line with other studies that conclude that symptomatic SARS-CoV-2 infection in pregnant women was associated with an increased risk of being admitted to the ICU [23,27].

### 4.2. Obstetric–Neonatal Outcomes and General Care

Hospitalized pregnant women with symptomatic SARS-CoV-2 were more likely to have not implemented a maternal labour companion or SSC and to have mother–infant separation after birth, as reported by another author [10]. We think that the fear of contagion, the small number of active professionals due to the scale of infections among professionals, and the ignorance of the disease could have influenced decision-making that we now know are not appropriate. The benefits of labour companionship are already known, with increasing spontaneous vaginal birth, decreasing caesarean birth and instrumental birth, and improving general satisfaction with childbirth [38,39]. Even though it may have a negative impact on our study outcomes, it was out of our sphere of analysis. Initially, separation was carried out in all cases driven by the Spanish Society of Neonatology recommendations at the time of the study [9,40]. Both practices, limited labour companionship and mother–infant separation, may have been influenced how the practice was modified as recommended by infection prevention and control (IPC) guidelines [41,42], driven by the fear of infection and the particularities of the healthcare system [43]. As reported by Coxon et al. [10], such measures may have been adopted in a context of uncertainty, even some medical paternalism [44] regarding the risks for the newborn infants or the professionals; in this regard, it seems that mother–infant separation occurred more often in the early days of the pandemic [10].

Although the results do not show statistically significant differences between groups of women, there is variation in the percentage of breastfeeding between groups. It is observed that symptomatic mothers have a lower percentage of follow-up, less SSC, and a higher percentage of mother–infant separation. All this can influence the results of breastfeeding [45].

In terms of neonatal outcomes, over 95% of infants born to SARS-CoV-2 positive mothers were in good general condition at birth, as with previous reports [28,46]. However, the risk of admission to the NICU (OR = 5.21) was higher among infants born to symptomatic mothers, in line with another report (RR = 3.13–6.03) [8]. This is consistent with the World Health Organization’s living systematic review, which concluded that SARS-CoV-2 infection was associated with increased risk of admission to the NICU for the infant [24,28].

Thus, although the vertical transmission of SARS-CoV-2 is possible, current data suggest that it remains uncommon with uncertainty about this event’s degree and the timing of this event [28,39,44,47]. Only 1.6% of newborn infants were infected in the first month of life, suggesting a lack of vertical transmission after birth in all the infants. Further, this observation would support the data from different studies indicating that horizontal transmission generally occurs in the home environment [28,46,48].

EBF at discharge was the most frequent type of feeding in both groups and persisted in all the symptomatic women that chose EBF after birth. The possibility of SARS-CoV-2 transmission through breast milk is unclear, and there is no evidence that the virus is viable and transmitted through breast milk [28]. Furthermore, although breastfeeding enables the transmission of immune mechanisms from the mother to the infant [49,50], and, thus, the benefits of breastfeeding may outweigh the potential risk of transmission [13,46], considerations were also required for women to maintain any recommended SARS-CoV-2 infection prevention measures as well as hygienic measures when breastfeeding [49,50,51].

### 4.3. Strengths and Limitations

We are aware that our study has some limitations. Firstly, not all the hospitals of the Valencian community were included in the study. Even though 8 of the 26 hospitals with the most significant number of annual childbirths in our region were represented, the sample size may be small, though we included all positive cases assisted in the participating centers. The magnitude of the OR obtained should be considered in light of the limited sample size, corresponding to obstetrical outcomes and general care. This could have led to overestimating the likelihood of these outcomes. Finally, the study was based on data obtained from electronic care records, with the usual potential for information bias, and the quality of the information included in the records. This bias may have been mitigated, as recorded variables were common to all participating centers, and the clinical records referred to general birth care. In addition, the use of registry data limited access to variables of interest such as body mass index, ethnicity, or economic status.

Despite these limitations, our study reflects the scant evidence on the possible differences in obstetric and neonatal outcomes and general care between symptomatic and asymptomatic women infected with SARS-CoV-2. Our study took place in the first year of the COVID-19 pandemic, whether the changes that occurred by the Spanish Ministry of Health and Spanish Medical Societies were introduced simultaneously in all the centers, and they could have influenced the results obtained [9]. The findings may inform recommendations for the general care of women in a future health crisis.

## 5. Conclusions

The symptomatic infected women are at increased risk of admission to the ICU and to have preterm births and NICU admissions of their newborns compared with asymptomatic women. Further, symptomatic infected women are increased risk of mother–infant separation, not early SSC, and lack of a labour companion compared with asymptomatic women. The incidence of SARS-CoV-2 infection among women giving birth was low, and no vertical transmission was recorded in any infants after birth.

## Figures and Tables

**Table 1:** Births and incidence of women with SARS-CoV-2 infection during the study.

Hospital	Total Births	Births in Women Infected with SARS-CoV-2	Incidence
H1	1288	18	14.0
H2	1090	7	6.4
H3	1308	7	5.4
H4	1182	21	17.8
H5	1256	14	11.1
H6	679	11	16.2
H7	920	8	8.7
H8	4160	44	10.6
TOTAL	11,883	130	10.9

**Table 2:** Demographic characteristics of women infected with SARS-CoV-2 in the study.

Study Variables	Women Infected with SARS-CoV-2		
	Asymptomatic (*n* = 103; 79.2%)	Symptomatic (*n* = 27; 20.8%)	*p* *
	Mean (SD)	Mean (SD)	
**Age**	31.5 (4.9)	33.9 (5.5)	0.049
	***n* (%)**	***n* (%)**	
**Country of origin**			0.287
Spain	64 (81.0)	6 (29.0)	
Central and South America	14 (70.0)	15 (30.0)	
Rest of EU countries	12 (80.0)	3 (20.0)	
Africa	9 (81.8)	2 (18.2)	
Asia	4 (80.0)	1 (20.0)	
**Hospital of birth**			
H8	34 (97.1)	1 (2.9)	0.112
H1	18 (100.0)	0 (0.0)	
H4	15 (71.4)	6 (28.6)	
H5	10 (71.4)	4 (28.6)	
H6	8 (72.7)	3 (27.3)	
H3	7 (70.0)	3 (30.0)	
H2	6 (37.5)	10 (62.5)	

* Chi-squared; severe acute respiratory syndrome coronavirus-2 = SARS-CoV-2; EU = European Union; H = Hospital.

**Table 3:** Obstetric–neonatal characteristics of the study sample.

Variable	Women Infected with SARS-CoV-2 (*n*/%)		
	Asymptomatic	Symptomatic	*p* *
	(103/79.2%)	(27/20.8%)	
	Mean (SD)	Mean (SD)	
**Gestational age (weeks)**	39.4 (1.4)	38.8 (2.2)	0.595
**Apgar 1 min**	9.63 (0.8)	9.56 (1.0)	0.694
**Apgar 5 min**	9.93 (0.4)	9.93 (0.4)	0.825
	***n* (%)**	***n* (%)**	
**Parity**			
Multiparous	52 (50.5)	13 (48.2)	0.829
Primiparous	51 (49.5)	14 (51.8)	
**Previous maternal disease**			
No	94 (91.3)	21 (77.8)	0.084
Yes	9 (8.7)	6 (22.2)	
**Gestational disease**			
No	88 (85.4)	19 (70.4)	0.089
Yes	15 (14.6)	8 (29.6)	
**Foetal disease**			
No	94 (91.3)	24 (88.9)	0.713
Yes	9 (8.7)	3 (11.1)	
**Start of labour**			
Elective caesarean section	5 (4.8)	1 (3.7)	0.09
Spontaneous	59 (57.3)	14 (51.9)	
Induced	39 (37.9)	12 (44.4)	
**COVID-related Maternal complications prior to labour**			
Asymptomatic	103 (100.0)	23 (85.2)	0.002
Respiratory distress	0 (0.0)	3 (11.1)	
Thrombotic stroke	0 (0.0)	1 (13.7)	
**Type of birth**			
Elective caesarean section	5 (4.9)	1 (3.7)	0.269
Urgent caesarean section	11 (10.7)	6 (22.2)	
Eutocic	75 (72.8)	15 (55.6)	
Instrumental birth	12 (11.6)	5 (18.5)	
**Cause of caesarean section (*n* = 23)**			
Other	10 (58.8)	6 (85.7)	0.366
NRFHR	7 (41.2)	1 (14.3)	
**Maternal complications COVID-related before discharge**			
No complications	101 (98.1)	23 (85.2)	0.002
Dyspnoea	0 (0.0)	1 (3.7)	
Respiratory distress	0 (0.0)	2 (7.4)	
Others	2 (1.9)	0 (0.0)	
Death	0 (0.0)	1 (3.7)	
**RT-PCR testing of newborn infant on day 1 of life**			
Positive	0 (0.0)	0 (0.0)	1
Negative	103 (100.0)	27 (100.0)	
**Mother required ICU admission prior to labour**			
No	103 (100.0)	25 (92.6)	0.042
Yes	0 (0.0)	2 (7.4)	
**Mother required ICU admission before discharge**			
No	101 (98.1)	25 (92.6)	0.042
Yes	2 (1.9)	2 (7.4)	
**Newborn infant required admission to NICU before discharge**			
No	88 (85.4)	16 (59.3)	0.005
Yes	15 (14.6)	11 (40.7)	
**Mother requiring emergency readmission after discharge in first 6 weeks**			
No	102 (99.0)	27 (100.0)	1
Yes	1 (1.0)	0 (0.0)	
**Newborn infant requiring emergency readmission after discharge in first 6 weeks**			
No	100 (97.1)	26 (96.3)	1
Yes	3 (2.9)	1 (3.7)	
**Reason for maternal readmission after discharge in first 6 weeks**			
Puerperal fever	1 (0.9)	0 (0.0)	1
No	102 (99.1)	27 (100.0)	
**Reason for newborn infant readmission after discharge in first 6 weeks**			
No reason	100 (97.0)	26 (96.3)	
Choking	1 (1.0)	0 (0.0)	1
Non-COVID-19 respiratory infection	0 (0.0)	1 (3.7)	
SARS-CoV-2 + (hospital admission)	1 (1.0)	0 (0.0)	
SARS-CoV-2 + (emergency room care)	1 (1.0)	0 (0.0)	

* chi-squared; severe acute respiratory syndrome coronavirus-2 = SARS-CoV-2; NRFHR = non-reassuring foetal heart pattern; RT-PCR = real-time polymerase chain reaction; ICU = Intensive Care Unit; NICU = Neonatal Intensive Care Unit.

**Table 4:** Obstetric–neonatal general care of the study participants.

Variable	Women Infected with SARS-CoV-2		
	Asymptomatic (*n*/%)	Symptomatic (*n*/%)	* *p*
	(103/79.2%)	(27/20.8%)	
**Labour companion**			0.028
No	37 (35.9)	16 (59.3)	
Yes	66 (64.1)	11 (40.7)	
**Late umbilical cord clamping**			0.125
No	44 (42.7)	16 (59.3)	
Yes	59 (57.3)	11 (40.7)	
**Early skin-to-skin contact**			0.029
No	21 (20.4)	11 (40.7)	
Yes	82 (79.6)	16 (59.3)	
**Mother-infant separation during hospital admission**			<0.001
No	89 (86.4)	16 (59.3)	
Yes	14 (13.6)	11 (40.7)	
**Reason for mother–infant separation**			<0.001
No separation	89 (86.4)	16 (59.3)	
Monitoring and control	2 (1.9)	2 (7.4)	
Other	5 (4.9)	9 (33.3)	
Protocol to date	7 (6.8)	0 (0.0)	
**Feeding at discharge**			0.450
Formula feeding (maternal decision)	12 (11.6)	5 (18.5)	
Formula feeding (medical recommendation)	1 (0.9)	3 (11.1)	
Exclusive breastfeeding	76 (73.8)	16 (59.3)	
Mixed feeding (maternal decision)	6 (5.8)	2 (7.4)	
Mixed feeding (medical recommendation)	8 (7.9)	1 (3.7)	
**Feeding at six weeks**			0.408
Formula feeding	26 (25.2)	9 (33.3)	
Exclusive breastfeeding	55 (53.4)	14 (51.6)	
Mixed feeding	22 (21.4)	4 (14.8)	

* chi-squared; SARS-CoV-2 = severe acute respiratory syndrome coronavirus-2.

**Table 5:** Obstetric–neonatal outcomes and general care of the participants.

	Women Infected with SARS-CoV-2							
		Asymptomatic		Symptomatic				
		(103/79.2%)		(27/20.8%)				
		*n*	%	*n*	%	^a^ *p*	^b^ OR	95% CI
**Obstetric outcomes**								
Preterm birth	<37 w	3	2.9	6	22.2	<0.001	9.52	2.2–41.2
	≥37 w	100	97.1	21	77.8			
Foetal growth restriction	No	102	99.3	26	96.3	0.304	3.92	0.2–64.8
	Yes	1	0.7	1	3.7			
Gestational diabetes	No	100	97.1	26	96.3	0.832	1.28	0.1–12.8
	Yes	3	2.9	1	3.7			
Induced hypertension in pregnancy	No	103	100.0	26	96.3	0.051	-	-
	Yes	0	0.0	1	3.7			
Caesarean section	No	87	84.5	20	74.1	0.208	1.90	0.7–5.2
	Yes	16	15.5	7	25.9			
Operative delivery	No	91	88.3	22	81.5	0.346	1.72	0.6–5.4
	Yes	12	11.7	5	18.5			
ICU admission	No	101	98.1	25	92.6	0.005	8.78	1.5–50.9
	Yes	2	1.9	2	7.4			
Maternal complications prior labour/during puerperium COVID-related: cardiac. neurologic, thrombotic, respiratory manifestations	No	103	100.0	26	96.3	0.057	-	-
	Yes	0	0.0	1	3.7			
Maternal death	No	103	100.0	26	96.3	0.051	-	-
	Yes	0	0.0	1	3.7			
**Neonatal outcomes**								
Small for gestational age	No	97	94.2	27	100.0	0.199	-	-
	Yes	6	5.8	0	0.0			
Large for gestational age	No	103	100.0	26	96.3	0.052	-	-
	Yes	0	0.0	1	3.7			
Apgar < 7 at 5 min	No	100	97.1	26	96.3	0.832	1.28	0.1–12.8
	Yes	3	2.9	1	3.7			
NICU admission	No	91	88.3	16	69.6	<0.001	5.21	1.9–13.8
	Yes	12	11.7	11	30.4			
SARS-CoV-2 infection	No	0	0.0	0	0.0	-	-	-
	Yes	0	0.0	0	0.0			
**General care**								
Mother–infant separation during hospital admission	No	89	86.4	16	59.3	0.005	4.03	1.5–10.4
	Yes	14	13.6	11	40.7			
Early skin-to-skin-contact	No	21	20.4	11	40.7	0.029	2.11	1.1–4.1
	Yes	82	79.6	16	59.3			
Late umbilical cord clamping	No	44	42.7	16	59.3	0.125	1.69	0.9–3.4
	Yes	59	57.3	11	40.7			
Labour companion	No	37	35.9	16	59.3	0.028	2.11	1.1–4.2
	Yes	66	64.1	11	40.7			
EBF at discharge	No	27	26.2	11	59.3	0.140	0.52	0.2–1.3
	Yes	76	73.8	16	40.7			
EBF at six weeks	No	48	46.6	13	48.1	0.866	0.94	0.4–2.2
	Yes	55	53.4	14	51.9			

^a^ Chi-squared; ^b^ OR = crude odds ratio; CI = confidence interval; SARS-CoV-2 = severe acute respiratory syndrome coronavirus-2; EBF = exclusive breastfeeding; ICU = intensive care unit; NICU = neonatal intensive care unit.

## Data Availability

Data are available upon reasonable request. All necessary data are supplied and available in the manuscript; however, the corresponding author will provide the dataset upon request. All data relevant to the study are included in the article.
